# HIV-1 Nef is released in extracellular vesicles derived from astrocytes: evidence for Nef-mediated neurotoxicity

**DOI:** 10.1038/cddis.2016.467

**Published:** 2017-01-12

**Authors:** A Sami Saribas, Stephanie Cicalese, Taha Mohseni Ahooyi, Kamel Khalili, Shohreh Amini, Ilker Kudret Sariyer

**Affiliations:** 1 Department of Neuroscience, Center for Neurovirology, Lewis Katz School of Medicine, Temple University, Philadelphia, 19140 PA, USA

## Abstract

Human immunodeficiency virus-associated neurological disorders (HANDs) affect the majority of AIDS patients and are a significant problem among HIV-1-infected individuals who live longer because of combined anti-retroviral therapies. HIV-1 utilizes a number of viral proteins and subsequent cytokine inductions to unleash its toxicity on neurons. Among HIV-1 viral proteins, Nef is a small protein expressed abundantly in astrocytes of HIV-1-infected brains and has been suggested to have a role in the pathogenesis of HAND. In order to explore its effect in the central nervous system, HIV-1 Nef was expressed in primary human fetal astrocytes (PHFAs) using an adenovirus. Our results revealed that HIV-1 Nef is released in extracellular vesicles (EVs) derived from PHFA cells expressing the protein. Interestingly, HIV-1 Nef release in EVs was enriched significantly when the cells were treated with autophagy activators perifosine, tomaxifen, MG-132, and autophagy inhibitors LY294002 and wortmannin suggesting a novel role of autophagy signaling in HIV-1 Nef release from astrocytes. Next, Nef-carrying EVs were purified from astrocyte cultures and neurotoxic effects on neurons were analyzed. We observed that HIV-1 Nef-containing EVs were readily taken up by neurons as demonstrated by immunocytochemistry and immunoblotting. Furthermore, treatment of neurons with Nef-carrying EVs induced oxidative stress as evidenced by a decrease in glutathione levels. To further investigate its neurotoxic effects, we expressed HIV-1 Nef in primary neurons by adenoviral transduction. Intracellular expression of HIV-1 Nef caused axonal and neurite degeneration of neurons. Furthermore, expression of HIV-1 Nef decreased the levels of phospho-tau while enhancing total tau in primary neurons. In addition, treatment of primary neurons with Nef-carrying EVs suppressed functional neuronal action potential assessed by multielectrode array studies. Collectively, these data suggested that HIV-1 Nef can be a formidable contributor to neurotoxicity along with other factors, which leads to HAND in HIV-1-infected AIDS patients.

## 

Human immunodeficiency virus-1 (HIV-1), the etiological agent of AIDS, not only wreaks havoc on the immune system,^1, 2, 3^ but also inflicts the central nervous system (CNS)^4^ leading to HIV-associated neurological disorders (HANDs).^5, 6, 7, 8, 9, 10^ Before combined anti-retroviral therapies (cARTs), HIV-1 infections were responsible for neuroinflammation leading to encephalitis (HIVE) as evidenced by astrocytosis, neuronal loss, activated microglia and infiltration of macrophages in to infected brains.^7, 11^ The introduction of cART reduced the neuronal damage inflicted by HIV infection.^12^ The paradigm of AIDS neuropathogenesis was changed to less severe forms of HAND such as mild neurocognitive disorder and impairments in neurocognition, but it did not completely prevent the more severe form of HAND, HIV-1-associated dementia (HAD).^7, 13^

HIV-1 infects CD4+ T cells first and then proceeds to invade the brain by infiltrating the blood–brain barrier (BBB), and exhibiting its devastating effects at later stages to develop HAND in nearly half of all AIDS patients.^14^ HIV-1 infection in the brain generally occurs in macrophages and microglia in a receptor-mediated manner, whereas a limited number of astrocytes are infected nonproductively.^15^ Despite the fact that neurons bear surface receptors, HIV-1 does not infect these cells. However, there are reports indicating the presence of HIV-1 nucleic acids in neurons obtained from some AIDS patients.^16, 17^ In addition, HIV-1 was found to infect neuronal cell lines such as SK-N-MC in a CD4-independent manner.^18^ In fact, human neurons are more prone to the toxic effects of HIV-1 viral proteins and, subsequently released cytokines and chemokines.^19^ HIV-1 regulatory proteins such as Tat and Vpr, and viral envelope protein gp120 are known to damage the BBB.^20^ HIV-1 infection is known to induce inflammatory cytokines such as TNF-alpha, IL-1 and IL6.^21, 22^ In this respect, HIV-1 viral proteins dysregulate several CNS processes, such as chemokine production, glutamate transport and cellular pathways, to cause neurotoxicity.^19^

There are indications that HIV-1 Nef also has a role in neuronal toxicity but the extent of its effects in the CNS remains unknown. Nef is one of HIV-1's auxiliary proteins with a molecular weight of 27–34 kD. It is myristylated on its amino terminus by post-translational modification.^23^ Expression of Nef occurs during early HIV-1 infection of cells including astrocytes,^24, 25^ macrophages and CD4+ T cells, and is released from these infected cells as a cargo protein in extracellular vesicles (EVs). Nef exhibits its toxic effects when it reaches its destination^26^ and can alter endosomal morphology^27^ leading to the accumulation of multivesicular bodies (MVBs) and lysosomes. MVBs are in turn released as EVs.^26^ HIV-1 Nef appears to be responsible for many events leading to neurological impairments in the HIV-1-infected brain such as neuronal degeneration by inducing IP-10 release,^28^ cytokine production and negatively affecting cellular pathways.^29, 30^ Neurotoxic effects of HIV-1 Nef were shown using recombinant Nef on human glial cells and neurons.^31^ Furthermore, animal studies revealed that HIV-1 Nef-induced neurocognitive impairments in rats.^32, 33^ The effect of HIV-1 infection on the brain depends on the subtype of virus. Interestingly, some AIDS patients exhibited the presence of HIV-1 DNA in their infected brains, while the others had no detectable viral DNA after autopsies.^34^ HIV-1 Nef modulates progression of AIDS in patients with HAD compared with those without and subtype D is most likely associated with HAD.^35^ Although the neurotoxicity of HIV-1 gp120 and Tat has been widely studied and better understood,^36^ the neurotoxicity of HIV-1 Nef is still not clear despite some recent reports. In this study, we elucidate the neurotoxic effects of HIV-1 Nef utilizing both EVs and by directly expressing this viral protein in primary human neurons.

## Results

### HIV-1 Nef is released in EVs derived from primary human astrocytes

**Figure 1:**
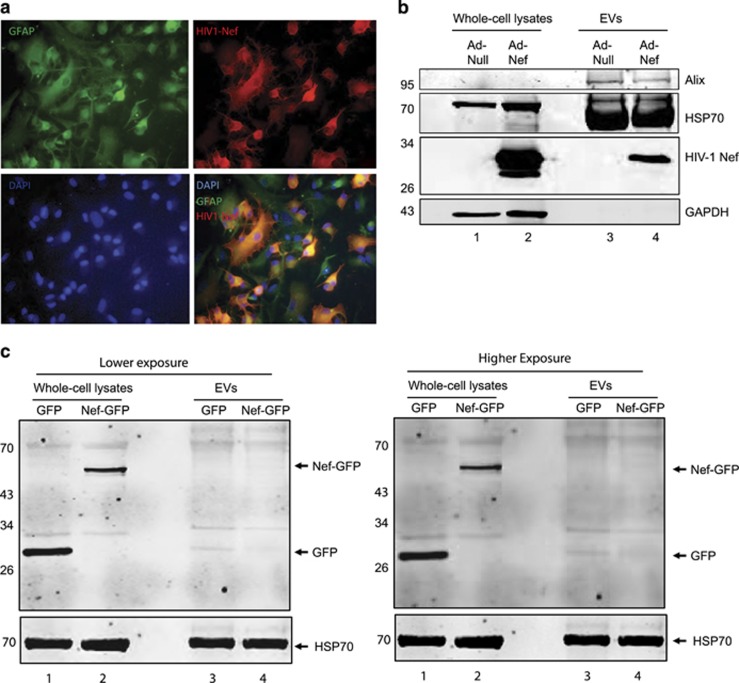
(**a**) Detection of HIV-1 Nef in EVs derived from PHFA. (**a**) Immunocytochemistry (ICC) of the PHFA cells transduced with Ad-Nef. The PHFA cells were grown in two-well chamber slides and transduced with Ad-Nef as described in Materials and Methods section. The cells were fixed, mounted and visualized by immunocytochemistry. First picture (upper left) shows GFAP – an astrocyte marker – distribution in astrocytes in green color. HIV-1 Nef expression in PHFA is shown in red color. Bottom left illustrates DAPI and bottom right is the illustration of merging GFAP, HIV-1 Nef and DAPI. (**b**) Western blot analyses of PHFA lysates and astrocytic EVs. PHFA cells were either transduced with Ad-Null or Ad-Nef for 24 h. Whole-cell protein lysates and EV lysates purified from growth media were separated on SDS-PAGE, transferred on a nitrocellulose membrane and analyzed by western blotting for the detection of Nef, HSP-70 and Alix using specific antibodies. GAPDH was also probed in the same membranes as a loading control. (**c**) Western blot analysis of PHFA lysates and astrocytic EVs. PHFA cells were transfected with an expression vector encoding either GFP or Nef-GFP for 48 h. Whole-cell protein lysates and EV lysates purified from growth media were separated on SDS-PAGE, transferred on a nitrocellulose membrane, and analyzed by western blotting for the detection of GFP, Nef-GFP and HSP-70. Both lower (left panels) and higher (right panels) exposures of the blots were shown and compared

In order to investigate whether HIV-1 Nef is released in EVs from astrocytes, PHFA cells were transduced with an adenovirus construct expressing Nef as described in Materials and Methods section. Cells were fixed and stained for glial fibrillary acidic protein (GFAP) and Nef by immunocytochemistry (Figure 1a). Ad-Nef-transduced cells were positive for the astrocyte marker GFAP in green. After 24 h post-transduction, EVs were prepared from the supernatants of PHFA cells using differential centrifugation as well as ExoQuick method as described in Materials and Methods section. Cell extracts from the transduced cells and the purified EVs were analyzed by western blot and compared with each other. Ad-Nef-transduced PHFA lysates had high levels of HIV-1 Nef protein as shown in the western blot (Figure 1b). EVs derived from these cells also contained HIV-1 Nef protein indicating that Nef protein is indeed associated with these EVs. We also analyzed the exosome-depleted astrocyte supernatants for HIV-1 Nef and found that there was no free Nef protein detectable (data not shown). Astrocytic EVs are also known to carry HSP-70.^37^ We have observed high levels of HSP-70 in both Null and Nef EVs. GAPDH was used as a loading control for cell lysates. Both Null and Nef-EVs were positive for Alix, which is an exosome marker. Alix was not at detection range in cell lysates most likely due to the low levels of this protein in cell lysates but more in EVs. It has been recently shown that crude EVs from Jurkat cells expressing Nef-EGFP did not incorporate Nef into the EVs.^38^ In order to test whether primary astrocytes could release Nef-EGFP in EVs, PHFA cells were transfected with an expression vector encoding either EGFP alone or Nef-EGFP. After 48 h post-transfection, EVs were prepared from the supernatants of PHFA cells using differential centrifugation. Cell extracts from the transduced cells and the purified EVs were analyzed by western blotting (Figure 1c). Interestingly, crude EV lysates from cells transduced with adenovirus expressing Nef-GFP contained only a trace amount of Nef-EGFP (Figure 1c, higher exposure) suggesting that EGFP fusion with Nef may interfere with Nef release in EVs.

### Role of autophagy in Nef release in EVs

**Figure 2:**
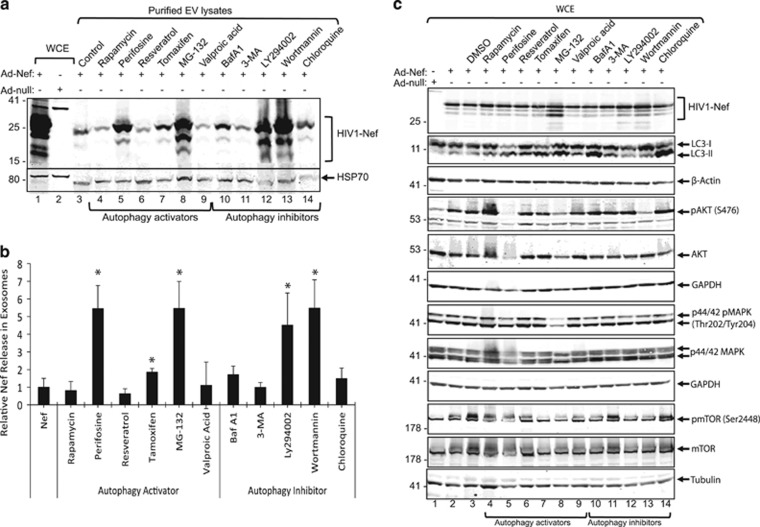
Effect of autophagy signaling on Nef release in EVs from PHA cells. (**a**) PHFA cells were transduced with Ad-Null and Ad-Nef constructs and treated with various autophagy activators and inhibitors as described in Materials and Methods section. EVs were purified from culture supernatants, lysed with TNN buffer containing 1% NP40 and analyzed by western blotting for the detection of HIV-1 Nef and HSP-70 proteins. In lanes 1 and 2, whole-cell protein lysates from PHFA cells transduced either with Ad-Nef or Ad-Null were loaded as positive and negative controls of Nef detection. (**b**) Protein bands from panel **a** on the western blot membranes were used to quantitate HIV-1 Nef levels and HSP-70 by ImageJ program and the relative values were plotted after normalizing Nef with HSP-70 using Excel program. The results are shown in bar graphs averages from the three independent experiments. (**c**) In parallel to culture supernatants, whole-cell protein lysates were also prepared from the same studies presented in panel **a** and processed by western blotting for the detection of HIV-1 Nef, LC3, pAKT (S476), total AKT, p44/42 MAPK (Thr202/Tyr204), total MAPK, pmTOR (S2448) and total mTOR expression levels. *β*-Actin, GAPDH and tubulin were also probed in same membranes as loading controls. *P<0.05

Several lines of evidence suggest a close interaction between autophagy pathways and the biogenesis and secretion of EVs.^54^ We have recently reported that expression of HIV-1 Nef compromises the autophagic pathways in primary human fetal astrocytes (PHFAs) by inducing autophagosome formation and blocking the assembly of autophagolysosomes.^39^ As shown in Figure 1b, Nef was readily detected in EV lysates obtained from PHFA cells expressing HIV-1 Nef. Therefore, we sought to investigate the possible role of autophagic signaling in Nef release in EVs. PHFA cells were plated in six-well tissue culture dishes and transduced with either Ad-null or Ad-Nef constructs. Cells were treated with several well-defined autophagy activators and inhibitors as described in Materials and Methods section. At 24 h post-treatments, culture media were collected and processed for EV purification by ExoQuick from System Biosciences (System Biosciences, CA, USA). As shown in Figures 2a and b, treatment of astrocytes with autophagy inhibitors perifosine (inhibitor of AKT phosphorylation), tomaxifen (estrogen receptor antagonist), MG-132 (proteasome inhibitor) and autophagy activators LY294002 (PI3 kinase inhibitor) and worthmannin (PI3 kinase inhibitor) caused a significant increase in Nef release in EVs. In parallel to the biochemical analysis of EVs, the possible impact of the treatments at indicated concentrations on cellular viabilities were also analyzed by MTT (3-(4,5-dimethylthiazol-2-yl)-2,5-diphenyltetrazolium bromide) assay. The MTT assay results indicated that none of the drugs at the concentrations used in panel A caused any significant toxicity (Supplementary Figure 1). Moreover, we have titrated the key autophagy activators (perifosine and MG-132) and autophagy inhibitors (LY294002 and wortmannin) as low, moderate and high doses. As shown in Supplementary Figure 2, all the drugs showed dose-responsive induction of Nef release in EVs suggesting their specific impact on endosomal biogenesis. In parallel to the culture media for EV purification presented in Figure 2a, cells were also harvested for whole-cell protein extraction and analyzed by western blotting. As shown in Figure 2c, HIV-1 Nef expression was mostly consistent with all treatments except MG-132, which caused a slight increase in total Nef protein levels in astrocytes (lane 8) suggesting that Nef turnover may be regulated by proteasomal degradation. Interestingly, Nef expression in astrocytes caused a significant increase in pAKT (S476) and mTOR (S2448) phosphorylation that was suppressed by perifosine, MG-132 and wortmannin treatments. LC3-I and its lipidated form LC3-II levels were also analyzed to monitor autophagic flux in accordance with treatments. Interestingly, the subsequent increase in Nef release in EVs did not correlate with LC3-I lipidation and activation of LC3-II.

### EVs carrying HIV-1 Nef are taken up by primary human neurons

**Figure 3:**
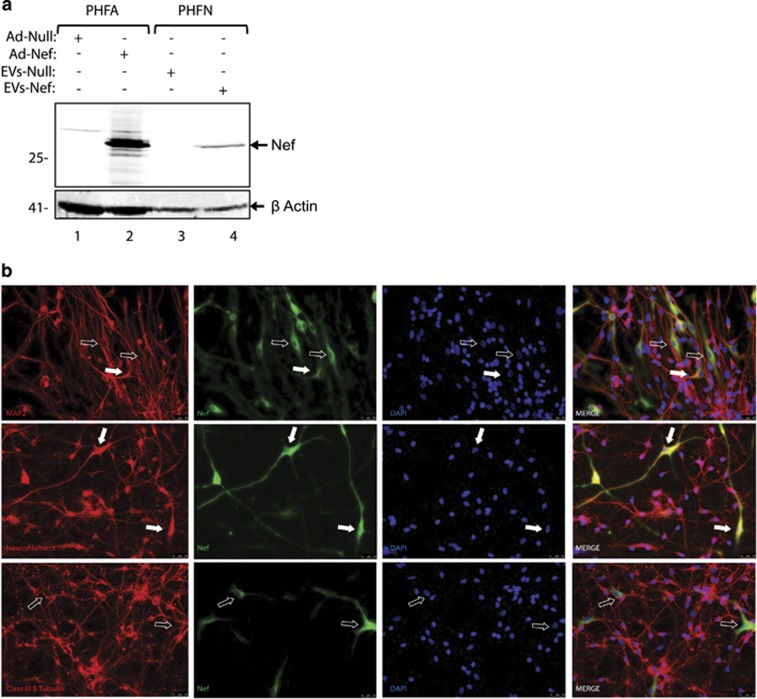
PHFA-derived EVs can deliver Nef to primary human neurons. (**a**) Western blot analysis of PHFN lysates treated with control and Nef-EVs. Whole-cell protein lysates from EV-treated PHFNs were prepared and resolved on SDS-PAGE (lanes 3 and 4). After transferring the gel on a nitrocellulose membrane, expression of HIV-1 Nef was analyzed by western blotting. Beta-actin was also probed in the same membranes as a loading control. In lanes 1 and 3, whole-cell lysates from PHFA cells transduced with Ad-Null and Ad-Nef were also loaded as negative and positive controls, respectively. (**b**) PHFNs were cultured in two-well chamber slides. Exosomes were purified from PHFA transduced with either Ad-Null or Ad-Nef and then used to treat neurons for 4 h. PHFNs were fixed at 48 h and processed by immunocytochemistry for the detection and labeling of MAP-2, neurofilament, class III *β*-tubulin and Nef. Open arrows point Nef positive, but MAP-2 or class III *β*-tubulin-negative cells. Closed arrows point cells with Nef double staining with MAP-2 and class III *β*-tubulin

Detection of HIV-1 Nef in EVs suggested that Nef could be delivered from astrocytes into the neurons by these EVs. To analyze possible uptake of Nef-containing EVs by neurons, PHFN cells were treated with EVs derived from PHFA cells either transduced with Ad-Null or Ad-Nef for approximately 48 h to ensure the uptake by neuronal cells. In order to analyze whether Nef is delivered into the neurons by EVs, cell lysates from PHFN cells treated with EVs were analyzed by western blotting. PHFN cells were harvested after extensive PBS washing followed by trypsinization to eliminate any surface-bound vesicles. As shown in Figure 3a, HIV-1 Nef protein was clearly detected in Nef EV-treated neuronal lysates suggesting that Nef was delivered into the neurons by EVs derived from PHFA cells expressing HIV-1 Nef. To gain more insight into the delivery of HIV-1 Nef into the neurons by EVs released from PHFA cells expressing Nef, immunocytochemistry was done on neurons treated with astrocyte-derived exosomes. PHFNs were cultured in two-well chamber slides and treated with Ad-Null or Ad-Nef EVs purified from PHFA, as described in Materials and Methods section. After 48 h of EV treatment, neurons were fixed, incubated with rabbit polyclonal anti-MAP-2, anti-neurofilament, anti-class III *β-*tubulin, double stained for monoclonal anti-Nef and processed by immunocytochemistry. As shown in Figure 3c, as expected, primary neurons showed high levels of MAP-2, neurofilament and class III *β*-tubulin expression (in red fluorescein color). On the other hand, several neurons showed immunoreactivity and typical cytoplasmic and perinuclear staining for HIV-1 Nef (in green fluorescein color) suggesting that Nef was delivered to neurons. Interestingly, neurons with Nef uptake showed decreased levels of MAP-2 and class III *β*-tubulin (open arrows) staining, suggesting a possible impact of Nef in neuronal cytoskeleton structure. Unlike MAP-2 and class III *β*-tubulin, neurons with Nef uptake showed normal neurofilament expression with perfect HIV-1 Nef colocalization (middle panel, closed arrows). These results suggest that HIV-1 Nef can be delivered by astrocytes to the neurons by EVs and may be associated with neurotoxicity.

### HIV-1 Nef causes axonal and neurite degeneration in primary human neurons

**Figure 4:**
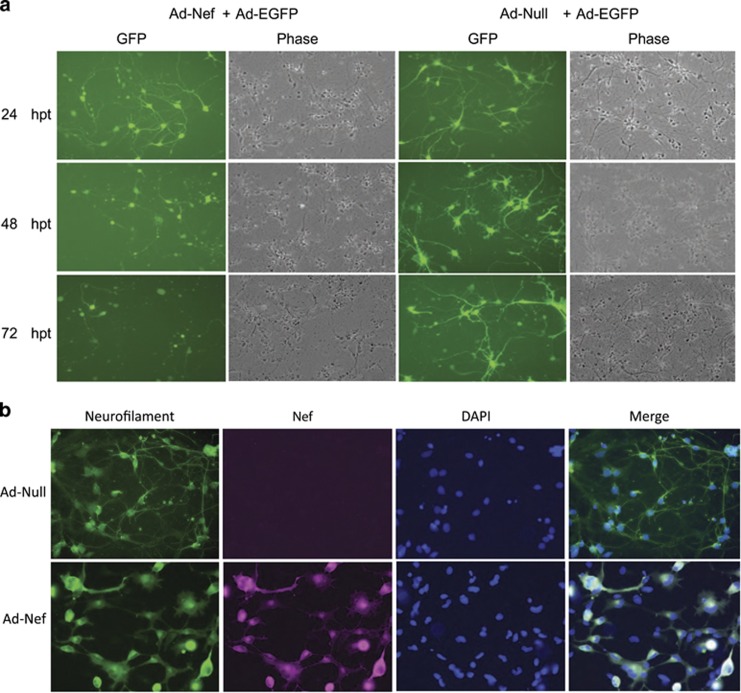
Nef expression in PHFN cells is detrimental. (**a**) PHFN cells were transduced with Ad-Null+Ad-EGFP or Ad-Nef+Ad-EGFP as described in Materials and Methods section. The expression of EGFP was followed for 3 days in live PHFN cultures by fluorescence microscopy. Left panel represents the effect of the HIV-1 Nef expression on neuronal architecture as monitored by EGFP expression as well as bright field microscopy. (**b**) ICC of the PHFNs transduced with Ad-Nef or Ad-Null, and effect of HIV-1-Nef on the neurofilament levels. The PHFN cells were seeded on two-well chamber slides, transduced with either Ad-Nef or Ad-Null, and fixed at 48 h post-transductions. The slides were probed with monoclonal HIV-1 Nef (SF2) and polyclonal neurofilament middle chain antibodies and visualized using a Leica Fluorescence Microscope. Top panel show the ICC of the Ad-Null, bottom panel shows the ICC of the Ad-Nef-transduced PHFNs. From left to right: neurofilament expression in green color, HIV-1-Nef expression in purple, DAPI staining in blue and merging of the neurofilaments, HIV-1-Nef and DAPI

We have tested the neurotoxic effect of HIV-1 Nef by intracellularly co-expressing the Nef protein with EGFP in neurons. For this purpose, we co-transduced PHFN with Ad-Nef and Ad-EGFP or Ad-Null and Ad-EGFP, and followed the expression of EGFP fluorescence over time. As EGFP is expressed throughout the transduced neurons, it serves as a tool to monitor structural integrity of neurons. Twenty-four hours post-transduction, EGFP expression was clearly seen distributed in neurons from the cell body to axons and dendrites (Figure 4a). In neurons transduced with Ad-Nef, cells started to display degeneration, whereas Ad-Null-treated neurons looked normal (Figure 3a). At 48 h post-transduction, most of the Ad-Nef-transduced PHFN cells exhibited the neurotoxic effect of the HIV-1 Nef losing most of their axons and dendrites and forming beaded structures, whereas the majority of the Ad-Null-transduced neurons remained unaffected. Finally, at 72 h post-transduction, cells transduced with Ad-Nef showed a total loss of axons and dendrites leaving only the cell body structures compared with Ad-Null control. In order to gain more insight into Nef-mediated neurotoxicity, neurofilament middle chain expression as a neuronal marker was also monitored in cells expressing HIV-1 Nef by immunocytochemistry. These experiments were conducted in slides by transducing primary neurons with adeno constructs; Ad-Nef or Ad-Null as described above and the slides were fixed at 48 h post-transduction. As shown in Figure 4b, Nef expression in primary neurons caused a marked change in neuronal morphology and resulted in re-distribution of neurofilament expression. These results have suggested that Nef expression in neurons is detrimental to neuronal processes, axons and dendrites.

### Effect of HIV-1 Nef expression on tau protein levels in PHFN and SH-SY5Y cells

**Figure 5:**
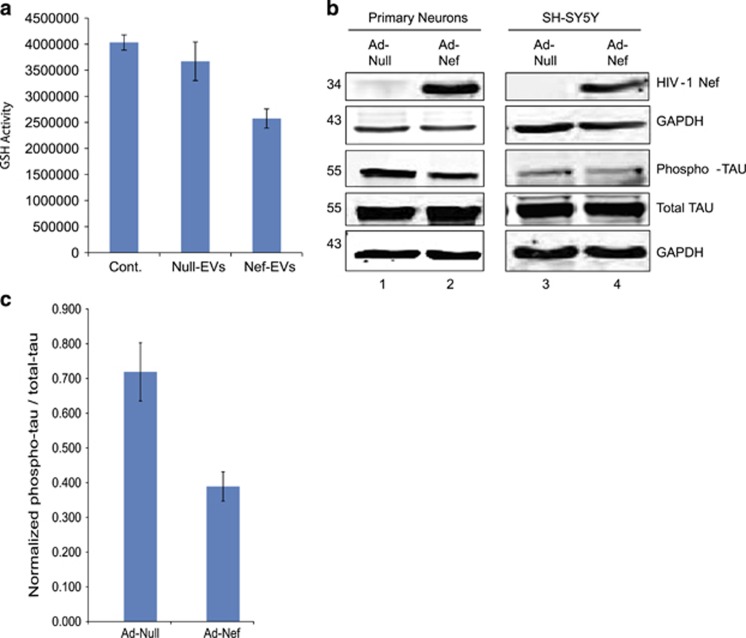
Effect of Nef on Tau and phospho-tau in primary neurons. (**a**) GSH-Glo Gluthatione assay for the EV-treated PHFNs. PHFN cells were grown in poly-d-lysine coated six-well plates in neuronal basal growth media at 37 ^o^C under 5% CO_2_ maintained by changing 1/2 media every 4 days. Nef and Null EVs were added to neurons as described before and incubated for 4 days before the harvest. PHFNs were harvested after trypsin treatment and GSH levels were measured according to the manufacturer's instructions. (**b**) Western blot analysis of the cell lysates obtained from the PHFN and SH-SY5Y neuroblastoma cells transduced with Ad-Nef or Ad-Null. Whole-cell lysates of the transduced PHFNs or SH-SY5Y cells were analyzed by western blotting for the detection of Nef, tau and phopsho-tau levels by using specific antibodies. GAPDH was also probed in the same membranes as a loading control. (**c**) Quantification of the proteins in Ad-Nef or Ad-Null-transduced PHFNs. Protein bands on the western blot membranes were used to quantitate tau and phospho-tau levels and loading controls, GAPDH by ImageJ program and the relative values were plotted after normalizing the tau and phospho-tau with GAPDH using Excel program. The results are shown in bar graphs averages from the three independent experiments

To analyze further whether Nef-EV treatment of neurons caused neurotoxicity, we utilized GSH-Glo Gluthatione assay. The test is based on conversion of a luciferin derivative into luciferin by GSH in a couple of enzymatic reactions where the luciferin produced is measured by luminescence, and GSH levels are proportional to luciferin. Changes in GSH levels in cells are indicative of oxidative stress that can lead to apoptosis and cell death. We found a significant decline of GSH levels in PHFN cells treated with Nef-EVs indicating that HIV-1 Nef may induce oxidative stress in primary human neurons (Figure 5a). To gain more insight into Nef-mediated neurotoxicity, the effect of HIV-1 Nef expression on total tau (t-tau) and phosphor-tau (p-tau) was analyzed in PHFN and SH-SY5Y neuroblastoma cells. PHFN and SH-SY5Y cells were transduced with either Ad-Null or Ad-Nef as described above. The whole-cell extracts obtained from the transduced cells were analyzed by immunoblotting. We observed that HIV-1 Nef is expressed both in PHFN and SH-SY5Y cells after Ad-Nef transduction; however, in these cells the expression levels of Nef protein was slightly less than that of primary human astrocytes (Figures 5b and c). HIV-1 Nef expression caused a significant decline in p-tau levels, whereas enhancing the t-tau levels in primary neurons (Figure 4a, compare lanes 1 and 2). When the p-tau/t-tau levels were compared with the control (Ad-Null), Nef-induced effect on p-tau was more significant (Figure 5c). HIV-1 Nef decreased the p-tau levels nearly 30%. In contrast, SH-SY5Y cells, which contain already lower p-tau levels were not affected by HIV-1 Nef expression. Both p-tau and t-tau levels were nearly identical in both Ad-Null and Ad-Nef-transduced cells (Figure 5b, compare lanes 3 and 4).

### Nef-EVs interfere with neuronal action potential assessed by multielectrode array (MEA) studies

In order to gain insight into the impact of Nef-carrying EVs on neurophysiological functions, we used MEA approach. Primary embryonic rat neurons (PERNs) were plated and cultured in MEA dishes as described in Materials and Methods section. Meanwhile, EVs were purified and characterized from culture supernatants of PHFA either transduced with Ad-Nef or Ad-Null. PERNs were treated with EVs released and obtained from same number of astrocytes over a period of 96 h. MEA extracellular action potential recordings were done at 0, 24, 48 and 96 h post-treatments. The recorded electrophysiological activities are shown for a 60-s time span (60–120 s); as the first 60 s is the time required for the MEA amplifier recovery after substation of the MEA. All recordings were performed at 37 °C and 7% CO_2_; the same condition employed to maintain the cells during the culturing period. As a reference, the activity of neurons was recorded right before starting the treatments (pretreatment condition of *t*=0 h). As depicted by Figure 6, a day after the treatment (*t*=24 h), the MEAs under the mentioned conditions experienced an increase of 55%, 33% and 115% compared with their basal pretreatment activity. This trend more or less holds for the controls in the following day, however, it is reversed for the Nef-treated cells. According to the recordings at *t*=48 h post treatment, the activities of controls increased by 25% compared with the preceding day. Nef-treated neurons showed a considerable decrease of 83%. The dropping of activity continues, and spiking activity in this culture totally disappears, whereas the number of recorded bursts of the negative and positive control returns to its basal activity within the succeeding 48 h.

## Discussion

HIV-1 does not infect human neurons, but instead it targets neurons by its proteins, Tat, Vpr, gp120 and Nef. Effects of HIV-1 Tat and gp120 on neurons have been studied extensively; however, toxicity caused by these viral proteins in the CNS is not completely understood.^40^ It has been shown that expression of HIV-1 Tat in astrocytes can lead to astrocytosis and subsequent death of neurons,^41^ and similar effects have also been reported with gp120-mediated neurotoxicity. Both Tat and gp120 appear to be responsible for serious neurotoxicity in HIV-1-infected brains.^40, 42, 43, 44^ Despite the gp120 and Tat, little is known about the possible impact of HIV-1 Nef on neurons. In our work, we have elucidated some of the neurotoxic effects of HIV-1 Nef on human neurons using a two-pronged approach: first to deliver the Nef protein to neurons by means of astrocytic exosomes and second to transduce the neuronal cells with Ad-Nef to express Nef protein intracellularly. Although the Nef gene was found in neurons isolated from some HIV-1-infected brains,^16, 17^ there was no evidence of Nef expression in these cells. Nevertheless, exposing neurons directly to HIV-1 Nef is crucial for understanding its neurotoxicity. HIV-1 Nef is primarily expressed in astrocytes,^45^ macrophages and microglia and exits these cells by means of EVs carrying a number of host biomolecules such as proteins, enzymes and RNA. We found that adenoviral Ad-Nef transduction of astrocytes not only generated high levels of HIV-1 Nef but also led to production of EVs carrying HIV-1 Nef. EVs have sometimes been ignored because it has been assumed that they are a mechanism of cellular garbage disposal. However, EVs have recently gained more attention after the discovery of their role in cell-to-cell communication^46, 47^ and cancer proliferation.^48^ In the CNS, a majority of cells, such as astrocytes, neurons, oligodendrocytes and microglia, release EVs in order to setablish neuronal communication.^49^ EVs also contribute to neuropathology by delivering the toxic form of proteins in, for example, Alzheimers' and Parkinson's diseases.^50, 51^ However, the EV machinery is also vulnerable to viral infections. A recent study demonstrated that HIV-1 exploits EVs to corrupt cell-to-cell communication in order to favor its spread in infected cells.^52^ HIV-1 Nef-containing EVs are released from the infected cells such as astrocytes and macrophages.^53^ These Nef-containing EVs can reach neurons where they deliver Nef's toxic effect. We chose human astrocyte-derived EVs to study Nef neurotoxicity, as the astrocytes are known to release EVs to support neurons. Our second approach was intracellular expression of Nef in neurons that exhibits this viral protein's ultimate neurotoxicity.

Several lines of evidence suggest that autophagy and secretion of EVs are coordinated mechanisms.^54^ We have recently reported that expression of HIV-1 Nef compromises the autophagic pathways in PHFAs by inducing autophagosome formation and blocking the assembly of autophagolysosomes.^39^ Here, we studied possible role of autophagy signaling in Nef release associated with EVs. Interestingly, autophagy inhibitors Perifosine (inhibitor of AKT phosphorylation), tomaxifen (estrogen receptor antagonist), MG-132 (proteasome inhibitor) and autophagy activators LY294002 (PI3 kinase inhibitor) and worthmannin (PI3 kinase inhibitor) caused a significant increase in Nef release in EVs. These results clearly provided evidence that autophagy pathways influenced by HIV-1 Nef may contribute to EV biogenesis and release of Nef in EVs from astrocytes. The exact role of autophagy in EV biogenesis and role of HIV-1 Nef in molecular crosstalk between autophagy and EV biogenesis remained to be determined.

HIV-1 Nef was readily detected in the EV-treated neurons, which was surprising and indicates that Nef delivery by EVs is sufficient for it to accumulate and be detected by immunoblotting. Our immunocytochemistry data also indicated that Nef EVs were taken up by human neurons. Using EVs to deliver Nef to the neurons also resulted in increased oxidative stress as measured by a decrease in GSH levels, an indication of neurotoxicity. We noticed significant neurotoxicity by expressing HIV-1 Nef in primary human neurons. Axonal and neurite degeneration is significantly higher in the presence of HIV-1 Nef in these cells indicating the toxic effect of the viral protein. Prolonged exposure to Nef completely destroyed nearly all axons and dendrites in the transduced cells. Furthermore, significant changes were also observed in tau proteins. Our finding shows that intracellular expression of HIV-1 Nef in primary neurons enhanced the t-tau while downregulating the p-tau levels. The relationship between HIV-1 infection, and t-tau and p-tau proteins in the brain are complex and not completely understood. Anthony *et al.*^55^ have reported that HIV-1-infected subjects have elevated levels of p-tau in particular in HAART-treated patients suggesting Alzheimers' like neuropathology. Gisslén *et al.*^56^ have analyzed t-tau and p-tau levels in 86 HIV-1+subjects including 21 of them with AIDS dementia complex (ADC) in a cross-sectional study demonstrating that while some ADC and HIV-1+infected patients had high t-tau in their CSF, there was no increase in p-tau levels unlike those seen in Alzheimers' patients. Interestingly, a recent study showed that amyotrophic lateral sclerosis patients have low levels of p-tau in their CSF and these low levels are correlated with neurocognitive dysfunction seen in this disease and p-tau/t-tau can be used as a biomarker for the disease progression. These results are intriguing as we also observed low p-tau in HIV-1 Nef-expressing primary neurons. In our case, the neurons are of fetal origin and not myelinated, therefore they do not represent adult human neurons. In SH-SY5Y neuroblastoma cells, however, we did not see any change in p-tau or t-tau levels. As these cells are transformed, the pathways governing the tau pathology could be different and therefore not affected by HIV-1 Nef. Possible impact of Nef-EVs on neuronal electrophysiology was also analyzed by MEA studies. Consistent with neurotoxicity assays, Nef-EVs interfered with neuronal action potential assessed by MEA studies.

HANDs manifest itself at many different levels in neuronal activities, such as deficiencies in cognition, motor functions and behavioral abnormalities, which are somewhat similar to other neurological diseases. Nearly half of the HIV-1 infection results with HAND. Overall, our results have suggested that EVs derived from primary astrocytes can deliver Nef into the neurons and contribute to the neurotoxicity associated with HAND development in patients infected with HIV-1.

## Materials and methods

### Ethics statement

All samples were obtained and utilized in accordance with Temple University Human Subjects Protections and the approval of the institutional review board.

### Plasmids and adenovirus constructs

Ad-Nef was constructed by cloning the HIV-1 Nef (SF2) gene from pcDNA3.1-SF2-Nef (NIH-AIDS reagent program, Germantown, MD, USA) into pDC515 (Ad-5) and propagated in HEK293A cells before purification using Opti-Prep gradient. Purified Ad-Nef had a titer of 1 × 10^9^ p.f.u./ml. To construct recombinant adenoviral vector harboring EGFP, EGFP cDNA was cloned in the adenovirus-shuttle plasmid pDC515 under the control of the murine cytomegalovirus promoter (purchased from Microbix Inc., Ontario, Canada). Adeno-EGFP recombinant shuttle containing EGFP sequence was transfected into HEK-293 cells with pBHGfrt (del) E1, 3FLP, and a plasmid that provides adenovirus type 5 genome deleted in E1 and E3 genes. Plaques of recombinant adenovirus arising as a result of frt/FLP recombination were isolated, grown and purified by cesium chloride density equilibrium banding. Empty shuttle plasmid, pDC515, was used to construct control Ad-Null, a virus without a transgene (Barrero *et al.*, 2013).^57^ The titers for Ad-.EGFP and Ad-Null were 3 × 10^10^ p.f.u./ml. Nef-EGFP construct was a kind gift from Dr. Michael D Powell (Campbell *et al.*, 2008).^58^

### Reagents

Antibodies were obtained from the following sources: anti-Nef (SF2), EH1 from NIH-AIDS Reagent Program, anti-GAPDH and anti-HSP-70 from Santa Cruz (Dallas, TX, USA), anti-tau from Santa Cruz and anti-PHF-tau from Pierce/Thermo Fisher, Inc., (Waltham, MA, USA), anti-beta tubulin class III from Sigma-Aldrich (St. Louis, MO, USA), anti-Alix Cell Signaling, Inc. (Beverly, MA, USA), LC3B from Sigma-Aldrich, *β*-tubulin from LI-COR, Odyssey (Lincoln, NE, USA), anti-neurofilament medium chain from Cell Signaling, Inc., anti-pAKT (Ser476), anti-AKT, p44/42 MAPK (Thr202/204), anti-MAPK, pmTOR (Ser2448), and anti-mTOR were purchased from Cell Signaling Technology (Danvers, MA, USA). Mammalian protease inhibitors were obtained from Sigma-Aldrich. Bradford reagent was from BioWorld (Dublin, OH, USA). GSH-Glo Glutathione Assay kit was purchased from Promega Corp. (Madison, WI, USA).

### Cell culture

Primary human fetal neurons (PHFN) were obtained in six-well plates or two-chamber slides from the Comprehensive Neuro-AIDS Center (CNAC) tissue culture core at Temple University Lewis Katz School of Medicine. Cells were maintained in Neurobasal medium (Gibco) containing B-27 supplement (Gibco/Thermo Fisher Inc. Waltham, MA, USA), 0.05% GlutaMAX (Gibco) and gentamicin (10 *μ*g/ml) at 37 ^o^C under 5% CO_2_. Half of the neuronal growth medium was replaced with fresh medium every 3–4 days. PHFAs were also obtained from CNAC tissue culture core. Cells were maintained in astrocyte growth media (Dulbecco's modified Eagle's medium (DMEM): F12 medium supplemented with 10% fetal bovine serum, 10% GlutaMAX, insulin and gentamicin (10 *μ*g/ml)) at 37 ^o^C under 5% CO_2_ atmosphere. The growth medium was changed every 3–4 days. PHFA were plated at densities of 2 × 10^6^ cells/100 mm dish for adenovirus transduction experiments. SH-SY5Y neuroblastoma cells were obtained from American Tissue Culture Collection (ATCC, Manassas, VA, USA, CRL-2266). They were plated in the poly-d-lysine-coated six-well dishes and maintained in astrocyte growth medium (described above) at 37 ^o^C under 5% CO_2_ atmosphere.

### Purification of the EVs (exosomes) from PFHA

Supernatants were obtained either from the un-transduced PHFA or PHFA transduced with Ad-Nef or Ad-Null. EVs were purified from these supernatants utilizing either ExoQuick (System Biosciences (SBI)) or differential centrifugation. In the ExoQuick method, 5 ml supernatant obtained from the Ad-Null or Ad-Nef-transduced PFHA cells was centrifuged at 3000 r.p.m. (Eppendorf Centrifuge, Eppendorf North America, Hauppauge, NY, USA, 5804R) for 30 min at 4 ^o^C, then treated with 1 ml ExoQuick reagent by gently mixing in a 15 ml sterile culture tube. The mixture was allowed to precipitate EVs overnight at 4 ^o^C. On the next day, the precipitated EVs were recovered by centrifugation at 3000 r.p.m. for 30 min at 4 ^o^C (Eppendorf Centrifuge, 5804R). The liquid phase was removed and tubes centrifuged briefly to collect the remaining liquid that was carefully removed by pipetting without disturbing the pellet. Finally, the pellets were gently rinsed with 1 ml cold 1 x PBS to remove the remnants of the reagent. The white colored exosome pellet obtained using ExoQuick was resuspended in 1 x PBS buffer. The exosome solution was either used directly or stored at −30 ^o^C. In the differential centrifugation method,^59^ approximately 30 ml supernatants from the transduced PHFA supernatants were first centrifuged at 3000 × g for 30 min at 4 ^o^C (Eppendorf Centrifuge, 5804R) to clear cell debris followed by a centrifugation at 10 000 × *g* for 30 min at 4 ^o^C (HB-6 rotor, Sorval Centrifuge, RC6+, Thermo Scientific) followed by filtration (Corning Incorp., NY, USA). At this step, clear supernatants were either stored at 4 ^o^C or being proceeded for ultracentrifugation. Ultracentrifugation was performed at 100 000 × *g* either using SW55Ti rotor at 35 000 r.p.m. for 3 h or SW24 at 24 000 r.p.m. for 4 h in a Beckman Ultracentrifuge. The EV pellets at this stage were small and had a transparent appearance with brownish color. After centrifugation, the tubes were inverted to remove the remaining liquid then the pellets were resuspended in 1 x PBS buffer. The EVs prepared by differential centrifugations are very similar to those obtained using ExoQuick method as determined by immunoblotting. The resuspended EV solutions were either used immediately or stored at −30 ^o^C until use. Protein contents of the EVs were determined by Bradford Protein Assay.

### Treatment of PHFA cells with autophagy inducers and inhibitors

PHFA cells were cultured in 60 mm tissue culture dishes (800 000 cells per dish). Cells were transduced with either Ad-Null or Ad-Nef and treated with autophagy activators rapamycin (50 nM), perifosine (50 *μ*M), resveratrol (75 *μ*M), tomaxifen (5 *μ*M), MG-132(10 *μ*M), valproic acid (0.6 mM) and autophagy inhibitors Baf-A1 (10 nM), 3-MA (10 mM), LY294002 (50 *μ*M), wortmannin (2 nM) and chloroquine (50 *μ*M). All chemicals and drugs were purchased from InvivoGen, San Diego, CA, USA. Supernatants were obtained either from the un-transduced PHFA or PHFA transduced with Ad-Nef or Ad-Null at 48 h post-transduction and 36 h post-treatments. EVs were purified from these supernatants utilizing ExoQuick (System Biosciences (SBI)), lysed in TNN buffer containing 1% NP40, 30 *μ*g total EV lysates from each treatment were separated on SDS-PAGE, and analyzed by western blotting for the detection of HIV-1 Nef and HSP-70. In parallel to the purification of EVs from culture supernatants, whole-cell lysates were also collected from the same studies, separated on SDS-PAGE, and analyzed by western blotting for the detection of HIV-1 Nef, LC3, pAKT(Ser476), total AKT, p44/42 MAPK, total MAPK, pmTOR (Ser2448) and total mTOR.

### Treatment of PHFN with astrocyte-derived EVs

PHFN were grown in 2 ml neurobasal growth media (Gibco) in six-well plates or in two-well chamber slides at 37 ^o^C under 5% CO_2_. For treatments, Null or Nef-EVs were first diluted and gently suspended in 1 × PBS to prevent self-aggregation. Then half of the neuronal growth medium was removed from the cell culture and aliquots of diluted EVs solutions were added to reach a final total protein concentration of approximately 20 *μ*g total proteins per well. The control cells were treated with same volume of 1 × PBS. The cells were then incubated at 37 ^o^C for 1 h followed by addition of 1 ml fresh neurobasal growth medium to each well. The EV-treated neurons were incubated 48 h at 37 ^o^C under 5% CO_2_ before harvesting for whole-cell protein extraction.

### Immunocytochemistry for Nef uptake by neurons

PHFNs were cultured in two-well chamber slides (500 000 cells per well). PHFNs were treated with Null or Nef-EVs by adding 20 *μ*g EVs in PBS (purified from PHFA media) to 400 *μ*l Opti-MEM and incubating at 37 ^o^C for 4 h. Following EV incubation, Opti-MEM was removed, and neurons were supplemented with neuronal media for 48 h. After incubation, neurons were fixed with cold acetone:methanol (50 : 50) for 1 min, washed three times with PBS, then, blocked with 10% BSA in PBS for 1 h, and incubated with rabbit polyclonal anti-MAP-2, anti-neurofilament, anti-class III *β-*tubulin, and monoclonal anti-Nef antibodies. Primary antibody dilutions were 1:300 in 5% BSA overnight at 4 °C with gentle rocking. After primary antibody incubation, cells were washed three times with PBS and then incubated in a secondary solution containing 1500 FITC mouse secondary and 1:500 rhodamine rabbit secondary, in 5% BSA for 2 h. Wells were then washed five times with PBS, and mounted with Vectashield mounting solution containing DAPI. Then glass coverslips were added before imaging on a Leica Fluorescence Microscope. (Leica Biosystems Inc., Buffalo Grove, IL, USA).

### Preparation of the PHFN and PHFA cell lysates, and SDS-PAGE/western blotting

The PHFA lysates were prepared 24–48 h after adenovirus transduction using TNN buffer with 1% NP40 supplemented with mammalian protease inhibitors. For 10% SDS-PAGE/western blot, 40 *μ*g proteins per well were used. The PHFN lysates were prepared as follows. At the end of incubations, cells were washed in PBS and harvested by trypsinization. PHFN were then lysed in TNN buffer with 1% NP40 supplemented with mammalian protease and phosphatase inhibitors, 0.4 mM NaF and 2 mM Na_3_VO_4._ The protein concentrations were determined by Bradford Protein Quantification assay. Protein samples were first denatured in SDS loading dye, heated at 95 ^o^C for 5 min, then separated on 10% SDS-PAGE. After electrophoresis, gels were transferred onto 0.45 *μ*m nitrocellulose membranes for 2 h at 250 mA, alternatively overnight at 40 mA in 1 × cold transfer buffer (100 mM TrisHCl, pH 7.4, 150 mM NaCl, 20% methanol). Membranes were blocked in either 1 x PBST buffer containing 10% milk or 1 x LI-COR blocking buffer at least 1 h at RT. The primary monoclonal antibodies, anti-HIV-1 Nef (sf2), anti-tau, anti-phospho-tau, anti-GAPDH, anti-alix, anti-beta tubulin class III, anti-HSP-70, anti-pAKT (Ser476), anti-AKT, p44/42 MAPK (Thr202/204), anti-MAPK, pmTOR (Ser2448) and anti-mTOR were diluted 1/1000 in buffer containing 5% milk and added to membranes where needed and were incubated overnight at 4 ^o^C with gentle shaking. After washing the membranes with 1 X PBST buffer three times, the secondary antibodies (LI-COR goat against rabbit 680 for polyclonal, LI-COR goat against mouse 800 for monoclonal) were added to the membranes and incubated for 45 min at RT followed by three times (5 min) washing with 1 X PBST buffer and a final wash with 1 X PBS buffer. Washed membranes were stored in 1 X PBS before scanning images on an ODISSEY Clx instrument. The intensity of each protein band was calculated using ImageJ (NIH) software (https://imagej.nih.gov/ij) and bar graphs were produced in Microsoft Excel program.

### GSH-Glo glutathione assay

The assay kit containing all necessary reagents was purchased from Promega Corp. PHFN were treated with Null and Nef-containing EVs separately as described above. After 4 days, PHFN were washed with PBS, trpysinized and resuspended in 200 *μ*l PBS buffer. The assay was carried out according to the manufacturer's guideline. Twenty microliters of Luciferin-NT and 20 *μ*l GST was first mixed with 2 ml GSH-Glo reaction buffer. From this mixture, 10 *μ*l resuspended neuronal samples were mixed with 100 *μ*l GSH reaction buffer containing Luciferin-NT and GST. The mixtures were incubated for 30 min at RT. Then 100 *μ*l GSH-Glo reagent were added to each tube followed by 15-min incubation at RT. The luminescence signals were measured in Zylux Femtomaster FB12 (Berthold Detection systems, Pforzheim, Germany). The averages of duplicate experiments were analyzed in Microsoft Excel and results plotted as bar graphs.

### MTT (3-(4,5-dimethylthiazol-2-yl)-2,5-diphenyltetrazolium bromide) assay for cell viability

PHFA cells were plated in six-well plates (2 × 10^5^), and treated with autophagy activators rapamycin (50 nM), perifosine (50 *μ*M), resveratrol (75 *μ*M), tomaxifen (5 *μ*M), MG-132(10 *μ*M), valproic acid (0.6 mM), and autophagy inhibitors Baf-A1 (10 nM), 3-MA (10 mM), LY294002 (50 *μ*M), wortmannin (2 nM) and chloroquine (50*μ*M). Twenty-four hours post-treatments, cells were incubated with 1 ml of MTT working solution (DMEM with 0.5 mg/ml MTT) for 2 h at 37 °C. The converted dye was solubilized with 1 ml acidic isopropanol (0.004 M HCL in isopropanol). Absorbance of the converted dye was measured at a wavelength of 570 nm with background subtraction at 650 nm.

### MEA recordings

All the recordings were performed using the MEA-1060 system (Multichannel Systems, Reutlingen, BW, Germany). The core component of this system is an MEA consisting of 60 titanium nitrite (TiN) electrodes covering a rectangular grid. Each electrode contains a circular TiN pad of 30 *μ*m diameter where the array spacing between each two neighboring electrodes varies in the range of 100–500 *μ*m. Before plating the cells, the MEAs underwent sterilization via applying 70% ethanol and then exposing the arrays to UV light for 60 min. As the MEA surface is originally hydrophobic, poly-d-lysine was used to hydrophilize the MEAs, as well as to provide a layer to enhance cell adhesion to the MEAs. To this end, poly-d-lysine (Sigma-Aldrich) was diluted in PBS with final concentration of 1 mg/ml and applied to the MEA surface for 2 h at 37 °C. In addition to poly-d-lysine, laminin (Invitrogen/Thermo Fisher, Inc., Waltham, MA, USA) was also introduced to the MEA surface (overnight at 37 °C) to promote long-lasting cellular adhesion (to culture cells for >10 days) and to improve the neural processes development. Once the MEAs were sterilized and coated, rat neural cells were plated on them with the average density of 5000 cells/mm^2^. Rat hippocampal cells were dissociated from the hippocampi of E18 prenatal rat embryos. After preparations, the suspended cells were directly placed onto the MEA surface where they settled adhered to the array within the next 3–4 h. For the best recording, quality neurons are required to stay and develop processes on the MEA for at least 12–14 days. During this period, neurons were maintained and fed using an appropriate medium in a regular manner. Experimental recordings were started when the cultures were 14 days old after the cells were treated by control and Nef-EVs obtained from PHFAs. After placing the MEAs on the amplifier, recordings were performed using the MC_Rack software (Reutlingen , BW, Germany) at a sampling frequency of 2000 kHz. To reduce the amount of noise while making sure to acquire the important information from neural spiking, an online built-in bandwidth filter was used throughout the recordings. The recorded spiking data (in *μ*V *versus* time) were transferred to the MATLAB environment for further offline analysis.

### Statistical analysis

All of the values presented on the graphs are given as means±S.E.M. ANOVA and unpaired Student's *t*-tests were used to analyze the statistical significance, and *P*-values <0.05 were considered statistically significant.

## Figures and Tables

**Figure 6:**
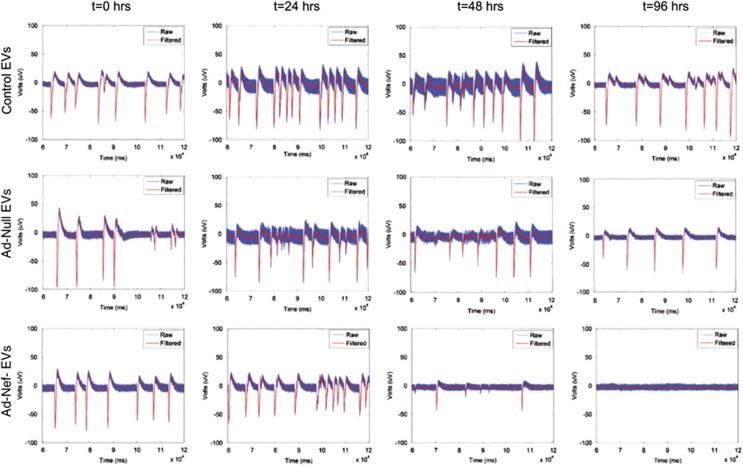
Extracellular action potential recordings of primary rat neurons treated with Nef-EVs by MEA. Rat hippocampal neurons were dissociated from the hippocampi of E18 prenatal rat embryos and placed onto the MEA dishes. For the best recording, quality neurons are required to stay and develop processes on the MEA for at least 12–14 days. Experimental recordings were started when the cultures were 14 days old after the cells were treated by control and Nef-EVs obtained from PHFAs. After placing the MEAs on the amplifier, recordings were performed using the MC_Rack software at a sampling frequency of 2000 kHz. The recorded spiking data (in *μ*V *versus* time) were transferred to the MATLAB environment for further offline analysis. Top row: untreated control at *t*=0, 24, 48 and 96 h. Middle row: Ad-Null EVs treated neurons at *t*=0, 24, 48 and 96 h. Bottom row: the Ad-Nef-EVs treated neurons at *t*=0, 24, 48 and 96 h

## References

[bib1] Pantaleo G, Fauci AS. Immunopathogenesis of HIV infection. Annu Rev Microbiol 1996; 50: 825–854. 8905100 10.1146/annurev.micro.50.1.825

[bib2] Moir S, Chun TW, Fauci AS. Pathogenic mechanisms of HIV disease. Annu Rev Pathol Mech Dis 2011; 6: 223–248. 10.1146/annurev-pathol-011110-130254 21034222

[bib3] Fraser C, Lythgoe K, Leventhal GE, Shirreff G, Hollingsworth TD, Alizon S. Bonhoeffer SVirulence and pathogenesis of HIV-1 infection: an evolutionary perspective. Science 2014; 343: 6177. 10.1126/science.1243727 PMC5034889 24653038

[bib4] Wiley CA, Schrier RD, Nelson JA, Lampert PW, Oldstone MB. Cellular localization of human immunodeficiency virus infection within the brains of acquired immune deficiency syndrome patients. Proc Natl Acad Sci USA 1986; 83: 7089–7093. 3018755 10.1073/pnas.83.18.7089 PMC386658

[bib5] Price RW, Brew B, Sidtis J, Rosenblum M, Scheck AC, Cleary P. The brain in AIDS: central nervous system HIV-1 infection and AIDS dementia complex. Science 1988; 239: 586–592. 3277272 10.1126/science.3277272

[bib6] Epstein LG, Gelbard HA. HIV-1-induced neuronal injury in the developing brain. J Leukoc Biol 1999; 65: 453–457. 10204573 10.1002/jlb.65.4.453

[bib7] Williams KC, Hickey WF. Central nervous system damage, monocytes and macrophages, and neurological disorders in AIDS. Annu Rev Neurosci 2002; 25: 537–562. 12052920 10.1146/annurev.neuro.25.112701.142822

[bib8] Kramer-Hämmerle S, Rothenaigner I, Wolff H, Bell JE, Brack-Werner R. Cells of the central nervous system as targets and reservoirs of the human immunodeficiency virus. Virus Res 2005; 111: 194–213. 15885841 10.1016/j.virusres.2005.04.009

[bib9] Lamers SL, Poon AF, McGrath MS. HIV-1 nef protein structures associated with brain infection and dementia pathogenesis. PLoS One 2011; 6: e16659. 21347424 10.1371/journal.pone.0016659 PMC3036659

[bib10] Desplats P, Dumaop W, Smith D, Adame A, Everall I, Letendre S et al. Molecular and pathologic insights from latent HIV-1 infection in the human brain. Neurology 2013; 80: 1415–1423. 23486877 10.1212/WNL.0b013e31828c2e9e PMC3662272

[bib11] Atwood WJ, Berger JR, Kaderman R, Tornatore CS, Major EO. Human immunodeficiency virus type 1 infection of the brain. Clin Microbiol Rev 1993; 6: 339–366. 8269391 10.1128/cmr.6.4.339 PMC358293

[bib12] Bryant AK, Ellis RJ, Umlauf A, Gouaux B, Soontornniyomkij V, Letendre SL et al. Antiretroviral therapy reduces neurodegeneration in HIV infection. AIDS 2015; 29: 323–330. 25686681 10.1097/QAD.0000000000000553 PMC4353640

[bib13] Zayyad Z, Spudich S. Neuropathogenesis of HIV: from initial neuroinvasion to HIV-associated neurocognitive disorder (HAND). Curr HIV/AIDS Rep 2015; 12: 16–24. 25604237 10.1007/s11904-014-0255-3 PMC4741099

[bib14] Price RW, Spudich SS, Peterson J, Joseph S, Fuchs D, Zetterberg H et al. Evolving character of chronic central nervous system HIV infection. Semin Neurol 2014; 34: 7–13. 24715483 10.1055/s-0034-1372337 PMC4120280

[bib15] Brack-Werner R. Astrocytes: HIV cellular reservoirs and important participants in neuropathogenesis. AIDS 1999; 13: 1–22. 10207540 10.1097/00002030-199901140-00003

[bib16] Torres-Muñoz JE, Núñez M, Petito CK. Successful application of hyperbranched multidisplacement genomic amplification to detect HIV-1 sequences in single neurons removed from autopsy brain sections by laser capture microdissection. J Mol Diagn 2008; 10: 317–324. 18556769 10.2353/jmoldx.2008.070074 PMC2438200

[bib17] Cantó-Nogués C, Sánchez-Ramón S, Alvarez S, Lacruz C, Muñóz-Fernández MA. HIV-1 infection of neurons might account for progressive HIV-1-associated encephalopathy in children. J Mol Neurosci 2005; 27: 79–89. 16055948 10.1385/JMN:27:1:079

[bib18] Li XL, Moudgil T, Vinters HV, Ho DD. CD4-independent, productive infection of a neuronal cell line by human immunodeficiency virus type 1. J Virol 1990; 64: 1383–1387. 2304148 10.1128/jvi.64.3.1383-1387.1990 PMC249262

[bib19] Rao VR, Ruiz AP, Prasad VR. Viral and cellular factors underlying neuropathogenesis in HIV associated neurocognitive disorders (HAND). AIDS Res Ther 2014; 11: 13. 24894206 10.1186/1742-6405-11-13 PMC4043700

[bib20] van de Bovenkamp M, Nottet HS, Pereira CF. Interactions of human immunodeficiency virus-1 proteins with neurons: possible role in the development of human immunodeficiency virus-1-associated dementia. Eur J Clin Invest 2002; 32: 619–627. 12190962 10.1046/j.1365-2362.2002.01029.x

[bib21] Tornatore C, Nath A, Amemiya K, Major EO. Persistent human immunodeficiency virus type 1 infection in human fetal glial cells reactivated by T-cell factor(s) or by the cytokines tumor necrosis factor alpha and interleukin-1 beta. J Virol 1991; 65: 6094–6100. 1920627 10.1128/jvi.65.11.6094-6100.1991 PMC250285

[bib22] Nuovo GJ, Gallery F, MacConnell P, Braun A. *In situ* detection of polymerase chain reaction-amplified HIV-1 nucleic acids and tumor necrosis factor-alpha RNA in the central nervous system. Am J Pathol 1994; 144: 659–666. 8160767 PMC1887241

[bib23] Geyer M, Fackler OT, Peterlin BM. Structure--function relationships in HIV-1 Nef. EMBO Rep 2001; 2: 580–585. 11463741 10.1093/embo-reports/kve141 PMC1083955

[bib24] Ranki A, Nyberg M, Ovod V, Haltia M, Elovaara I, Raininko R et al. Abundant expression of HIV Nef and Rev proteins in brain astrocytes *in vivo* is associated with dementia. AIDS 1995; 9: 1001–1008. 8527071 10.1097/00002030-199509000-00004

[bib25] Robichaud GA, Poulin L. HIV type 1 nef gene inhibits tumor necrosis factor alpha-induced apoptosis and promotes cell proliferation through the action of MAPK and JNK in human glial cells. AIDS Res Hum Retroviruses 2000; 16: 1959–1965. 11153078 10.1089/088922200750054684

[bib26] Lenassi M, Cagney G, Liao M, Vaupotic T, Bartholomeeusen K, Cheng Y et al. HIV Nef is secreted in exosomes and triggers apoptosis in bystander CD4+ T cells. Traffic 2010; 1: 110–122. 10.1111/j.1600-0854.2009.01006.x PMC2796297 19912576

[bib27] Madrid R, Janvier K, Hitchin D, Day J, Coleman S, Noviello C et al. Nef-induced alteration of the early/recycling endosomal compartment correlates with enhancement of HIV-1 infectivity. J Biol Chem 2005; 280: 5032–5044. 15569681 10.1074/jbc.M401202200

[bib28] Van Marle G, Henry S, Todoruk T, Sullivan A, Silva C, Rourke SB et al. Human immunodeficiency virus type 1 Nef protein mediates neural cell death: a neurotoxic role for IP-10. Virology 2004; 329: 302–318. 15518810 10.1016/j.virol.2004.08.024

[bib29] Percario ZA, Ali M, Mangino G, Affabris E. Nef, the shuttling molecular adaptor of HIV, influences the cytokine network. Cytokine Growth Factor Rev 2014piiS1359-6101: 00164–00166. 10.1016/j.cytogfr.2014.11.010 25529283

[bib30] Mukerji J, Olivieri KC, Misra V, Agopian KA, Gabuzda D. Proteomic analysis of HIV-1 Nef cellular binding partners reveals a role for exocyst complex proteins in mediating enhancement of intercellular nanotube formation. Retrovirology 2012; 9: 33. 22534017 10.1186/1742-4690-9-33 PMC3382630

[bib31] Trillo-Pazos G, McFarlane-Abdulla E, Campbell IC, Pilkington GJ, Everall IP. Recombinant nef HIV-IIIB protein is toxic to human neurons in culture. Brain Res 2000; 864: 315–326. 10802040 10.1016/s0006-8993(00)02213-7

[bib32] Mordelet E, Kissa K, Cressant A, Gray F, Ozden S, Vidal C et al. Histopathological and cognitive defects induced by Nef in the brain. FASEB J 2004; 18: 1851–1861. 15576488 10.1096/fj.04-2308com

[bib33] Chompre G, Cruz E, Maldonado L, Rivera-Amill V, Porter JT, Noel RJJr.. Astrocytic expression of HIV-1 Nef impairs spatial and recognition memory. Neurobiol Dis 2013; 49: 128–136. 22926191 10.1016/j.nbd.2012.08.007 PMC3530662

[bib34] Koppensteiner H, Brack-Werner R, Schindler M. Macrophages and their relevance in human immunodeficiency virus type I infection. Retrovirology 2012; 9: 82. 23035819 10.1186/1742-4690-9-82 PMC3484033

[bib35] Lamers SL, Salemi M, Galligan DC, Morris A, Gray R, Fogel G et al. Human immunodeficiency virus-1 evolutionary patterns associated with pathogenic processes in the brain. J Neurovirol 2010; 16: 230–241. 20367240 10.3109/13550281003735709 PMC2994721

[bib36] Mattson MP, Haughey NJ, Nath A. Cell death in HIV dementia. Cell Death Differ 2005; 12((Suppl 1)): 893–904. 15761472 10.1038/sj.cdd.4401577

[bib37] Sampey GC, Meyering SS, Asad Zadeh M, Saifuddin M, Hakami RM, Kashanchi F. Exosomes and their role in CNS viral infections. J Neurovirol 2014; 20: 199–208. 24578033 10.1007/s13365-014-0238-6 PMC4378677

[bib38] Luo X, Fan Y, Park IW, He JJ. Exosomes are unlikely involved in intercellular nef transfer. PLoS One 2015; 10: e0124436. 25919665 10.1371/journal.pone.0124436 PMC4412529

[bib39] Saribas AS, Khalili K, Sariyer IK. Dysregulation of autophagy by HIV-1 Nef in human astrocytes. Cell Cycle 2015; 14: 2899–2904. 26176554 10.1080/15384101.2015.1069927 PMC4825552

[bib40] Mocchetti I, Bachis A, Avdoshina V. Neurotoxicity of human immunodeficiency virus-1: viral proteins and axonal transport. Neurotox Res 2012; 21: 79–89. 21948112 10.1007/s12640-011-9279-2 PMC4089041

[bib41] Zhou BY, Liu Y, Bo Kim, Xiao Y, He JJ. Astrocyte activation and dysfunction and neuron death by HIV-1 Tat expression in astrocytes. Mol Cell Neurosci 2004; 27: 296–305. 15519244 10.1016/j.mcn.2004.07.003

[bib42] Ferrell D, Giunta B. The impact of HIV-1 on neurogenesis: implications for HAND. Cell Mol Life Sci 2014; 22: 4387–4392. 10.1007/s00018-014-1702-4 PMC4224299 25134912

[bib43] Fields JA, Dumaop W, Crews L, Adame A, Spencer B, Metcalf J et al. Mechanisms of HIV-1 Tat neurotoxicity via CDK5 translocation and hyper-activation: role in HIV-associated neurocognitive disorders. Curr HIV Res 2015; 13: 43–54. 25760044 10.2174/1570162x13666150311164201 PMC4455959

[bib44] Fields JA, Dumaop W, Elueteri S, Campos S, Serger E, Trejo M et al. HIV-1 Tat alters neuronal autophagy by modulating autophagosome fusion to the lysosome: implications for HIV-associated neurocognitive disorders. J Neurosci 2015; 35: 1921–1938. 25653352 10.1523/JNEUROSCI.3207-14.2015 PMC4315828

[bib45] Gray LR, Turville SG, Hitchen TL, Cheng WJ, Ellett AM, Salimi H et al. HIV-1 entry and trans-infection of astrocytes involves CD81 vesicles. PLoS One 2014; 9: e90620. 24587404 10.1371/journal.pone.0090620 PMC3938779

[bib46] Simons M, Raposo G. Exosomes--vesicular carriers for intercellular communication. Curr Opin Cell Biol 2009; 21: 575–581. 19442504 10.1016/j.ceb.2009.03.007

[bib47] Pant S, Hilton H, Burczynski ME. The multifaceted exosome: biogenesis, role in normal and aberrant cellular function, and frontiers for pharmacological and biomarker opportunities. Biochem Pharmacol 2012; 83: 1484–1494. 22230477 10.1016/j.bcp.2011.12.037 PMC7110994

[bib48] Greening DW, Gopal SK, Xu R, Simpson RJ, Chen W. Exosomes and their roles in immune regulation and cancer. Semin Cell Dev Biol 2015; 40: 72–81. 25724562 10.1016/j.semcdb.2015.02.009

[bib49] Frühbeis C, Fröhlich D, Kuo WP, Krämer-Albers EM. Extracellular vesicles as mediators of neuron-glia communication. Front Cell Neurosci 2013; 7: 182. 24194697 10.3389/fncel.2013.00182 PMC3812991

[bib50] Rajendran L, Bali J, Barr MM, Court FA, Krämer-Albers EM, Picou F et al. Emerging roles of extracellular vesicles in the nervous system. J Neurosci 2014; 34: 15482–15489. 25392515 10.1523/JNEUROSCI.3258-14.2014 PMC4228143

[bib51] Gupta A, Pulliam L. Exosomes as mediators of neuroinflammation. J Neuroinflammation 2014; 11: 68. 24694258 10.1186/1742-2094-11-68 PMC3994210

[bib52] Arenaccio C, Chiozzini C, Columba-Cabezas S, Manfredi F, Affabris E, Baur A et al. Exosomes from human immunodeficiency virus type 1 (HIV-1)-infected cells license quiescent CD4+ T lymphocytes to replicate HIV-1 through a Nef- and ADAM17-dependent mechanism. J Virol 2014; 88: 11529–11539. 25056899 10.1128/JVI.01712-14 PMC4178784

[bib53] Ali SA, Huang MB, Campbell PE, Roth WW, Campbell T, Khan M et al. Genetic characterization of HIV type 1 Nef-induced vesicle secretion. AIDS Res Hum Retroviruses 2010; 26: 173–192. 20156100 10.1089/aid.2009.0068 PMC2835390

[bib54] Baixauli F, López-Otín C, Mittelbrunn M. Exosomes and autophagy: coordinated mechanisms for the maintenance of cellular fitness. Front Immunol 2014; 5: 403. 25191326 10.3389/fimmu.2014.00403 PMC4138502

[bib55] Anthony IC, Ramage SN, Carnie FW, Simmonds P, Bell JE. Accelerated Tau deposition in the brains of individuals infected with human immunodeficiency virus-1 before and after the advent of highly active anti-retroviral therapy. Acta Neuropathol 2006; 111: 529–538. 16718349 10.1007/s00401-006-0037-0

[bib56] Gisslen M, Krut J, Andreasson U, Blennow K, Cinque P, Brew BJ et al. Amyloid and tau cerebrospinal fluid biomarkers in HIV infection. BMC Neurol 2009; 9: 63. 10.1186/1471-2377-9-63 PMC2807422 20028512

[bib57] Barrero CA, Datta PK, Sen S, Deshmane S, Amini S, Khalili K et al. HIV-1 Vpr modulates macrophage metabolic pathways: a SILAC-based quantitative analysis. PLoS One 2013; 8. 10.1371/journal.pone.0068376 PMC3709966 23874603

[bib58] Campbell TD, Khan M, Huang MB, Bond VC, Powell MD. HIV-1 Nef protein is secreted into vesicles that can fuse with target cells and virions. Ethn Dis 2008 18(2 Suppl 2): S2-14–9. PMC3418053 18646314

[bib59] Van Deun J, Mestdagh P, Sormunen R, Cocquyt V, Vermaelen K, Vandesompele J et al. The impact of disparate isolation methods for extracellular vesicles on downstream RNA profiling. J Extracell Vesicles 2014; 3: 24858. 10.3402/jev.v3.24858 PMC4169610 25317274

