# Supplementary material for: HIV-1 Nef is released in extracellular vesicles derived from astrocytes: evidence for Nef-mediated neurotoxicity
Source: Cell Death Dis. 2017 Jan 12;8(1):e2542–. doi: 10.1038/cddis.2016.467 (PMC5386374; doi:10.1038/cddis.2016.467)
Supplement: Supplementary Information [file cddis2016467x1.docx]

**SUPPLEMENTARY FIGURES**


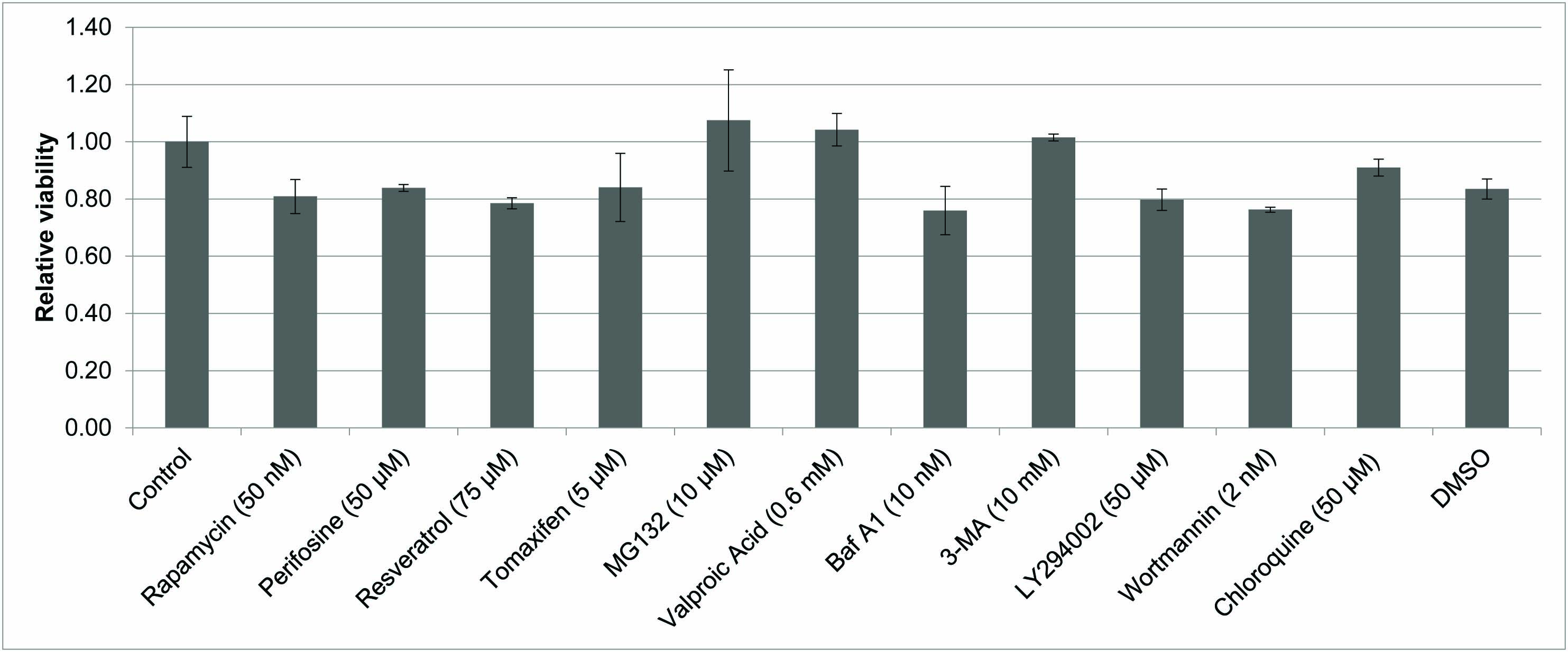


**Supplementary figure 1: Effect of autophagy inhibitors and activators on cellular viability of PHFA cells.** PHFA cells were plated in 6-well tissue culture dishes and treated with various autophagy activators and inhibitors at indicated concentrations for 24 hours. MTT (3-(4,5-Dimethylthiazol-2-yl)-2,5-diphenyltetrazolium bromide) assay was performed to determine possible cytotoxicity associated with treatments. Twenty four hours post-treatments, cells were incubated with 1 ml of MTT working solution (DMEM with 0.5 mg/ml MTT) for 2 hours at 37°C. The converted dye was solubilized with 1 ml acidic isopropanol (0.004 M HCL in isopropanol). Absorbance of the converted dye was measured at a wavelength of 570 nm with background subtraction at 650 nm. The readings were normalized to the control (untreated) and shown as relative viability. Data represents the mean of three independent experiments.


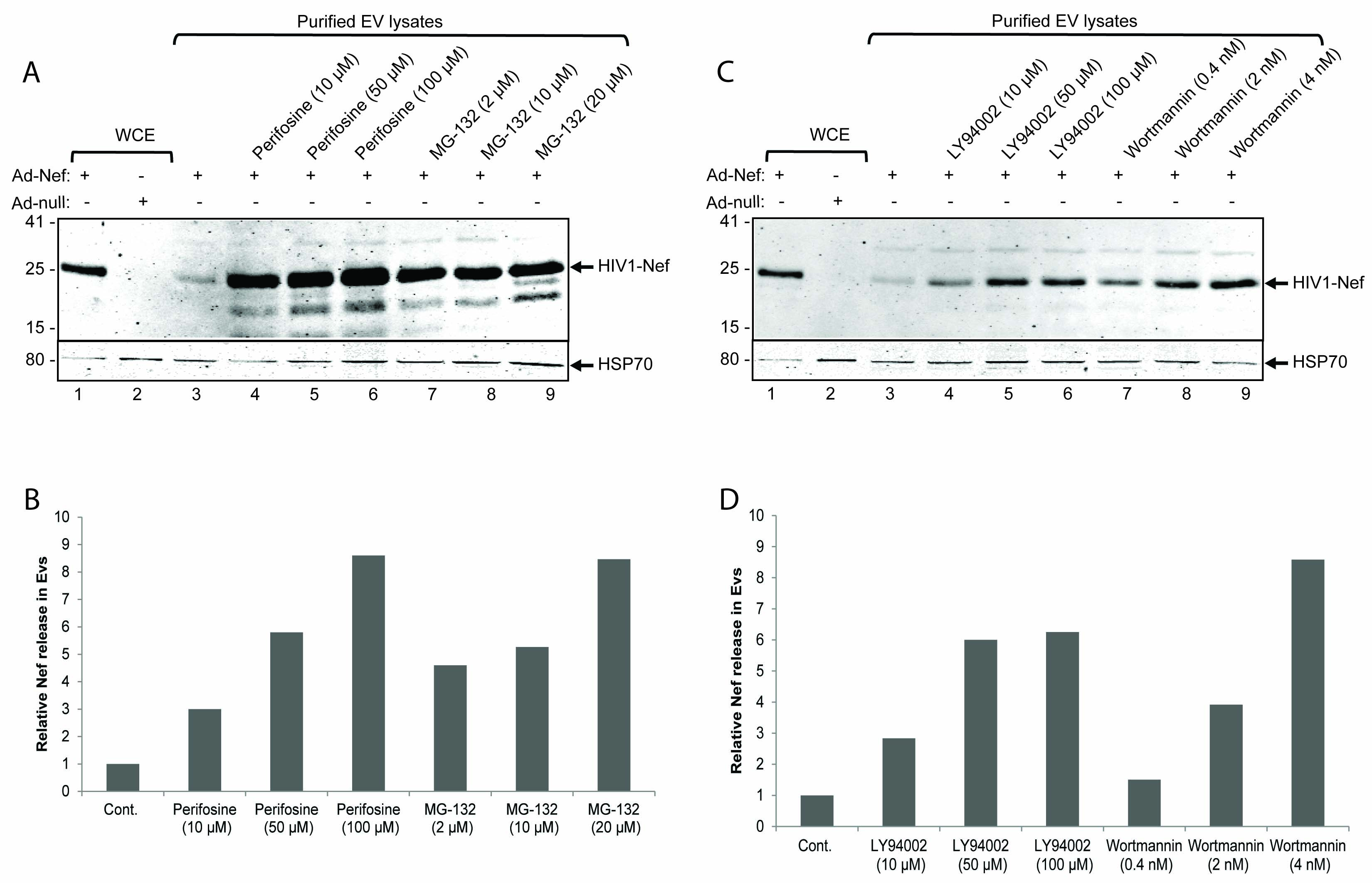


**Supplementary figure 2: Dose-responsive impact of autophagy inhibitors and activators on HIV1-Nef release in EVs. A.** PHFA cells were transduced with Ad-Null and Ad-Nef constructs and treated with increasing concentrations of autophagy activators perifosine (10, 50, and 100 µM) and MG-132 (2, 10, and 20 µM). EVs were purified from culture supernatants, lysed with TNN buffer containing 1% NP40, and analyzed by Western blotting for the detection of HIV-1 Nef and HSP70 proteins. In lane 1 and 2, whole cell protein lysates from PHFA cells transduced either with Ad-Nef or Ad-Null were loaded as positive and negative controls of Nef detection. B. Protein bands from panel A on the Western blot membranes were used to quantitate HIV-1 Nef levels by IMAGEJ program and the relative values were plotted. C. PHFA cells were transduced with Ad-Null and Ad-Nef constructs and treated with increasing concentrations of autophagy inhibitors LY94002 (10, 50, and 100 µM) and Wortmannin (0.4, 2, and 4 nM). EVs were purified from culture supernatants, lysed with TNN buffer containing 1% NP40, and analyzed by Western blotting for the detection of HIV-1 Nef and HSP70 proteins. In lane 1 and 2, whole cell protein lysates from PHFA cells transduced either with Ad-Nef or Ad-Null were loaded as positive and negative controls of Nef detection. D. Protein bands from panel C on the Western blot membranes were used to quantitate HIV-1 Nef levels by IMAGEJ program and the relative values were plotted.
